# Developing content for national population health surveys: an example using a newly developed sedentary behaviour module

**DOI:** 10.1186/s13690-019-0380-y

**Published:** 2019-12-04

**Authors:** Stephanie A. Prince, Gregory P. Butler, Karen C. Roberts, Pam Lapointe, Andrew M. MacKenzie, Rachel C. Colley, Maria Foley, Travis J. Saunders, Wendy Thompson

**Affiliations:** 1 Centre for Surveillance and Applied Research, Public Health Agency of Canada, 785 Carling Avenue, Ottawa, K1A 0K9 Canada; 2 Division of Cardiac Prevention and Rehabilitation, University of Ottawa Heart Institute, Ottawa, Canada; 3 Health Analysis Division, Statistics Canada, Ottawa, Canada; 4 Health Statistics Division, Statistics Canada, Ottawa, Canada; 5 Department of Applied Human Sciences, University of Prince Edward Island, Charlottetown, Canada

**Keywords:** Survey, Questionnaire, Population health, Sedentary behaviour

## Abstract

**Background:**

While physical (in) activity surveillance has grown and continues to grow globally, surveillance of sedentary behaviour is in its infancy. As surveillance evolves to meet the changing nature of these behaviours, there is a need for the development of national health survey questions to provide accurate and consistent measures over time. The development of national health survey content is a complex, detailed and often undocumented process. The objective of this paper is to outline the process that the Public Health Agency of Canada (PHAC) and Statistics Canada took in partnership with academic experts to develop a short, flexible, sedentary behaviour module for the Canadian Health Measures Survey (CHMS) and to provide an approach for the development of future survey content.

**Methods:**

Development of the module followed a multi-step process. The results of this paper describe this process and present a framework for content development.

**Results:**

Initially, PHAC and Statistics Canada analysts worked together to identify key content required for a potential survey module. Next, this work was formalized through a contract with academic experts, the scope included a: review of existing Canadian sedentary behaviour modules; literature review linking different sedentary behaviours to health outcomes; and, international scan of modules currently in use in large national health surveys and research. The key output from both review processes was recommendations for a short sedentary behaviour questionnaire module (International Sedentary Assessment Tool). These recommendations provided an evidence-informed basis for discussions about how to revise and update the CHMS sedentary behaviour questionnaire content. Qualitative testing was undertaken and a final module was developed using survey design best practices.

**Conclusions:**

Content volume in national surveys is limited due to demand to measure core content in addition to emerging health topics while keeping surveys as short as possible. Questions must therefore, be concise, valid/reliable, evidence-based, and developed using best practices. The paper describes the development process of a new survey module addressing the emerging area of sedentary behaviour for use in a national survey that may serve as a model for future population survey content development.

## Background

In 2012, the Lancet Physical Activity Series Working Group used data from the WHO global health observatory data repository to report on the global prevalence of physical inactivity [1]. While it is recognized that physical (in) activity surveillance has grown and continues to grow globally, surveillance of sedentary behaviour is in its infancy [1]. Relative to physical activity, the field of study around sedentary behaviour is young, and only in the past 10 years has it received a surge of attention [2]. Sedentary behaviour is often confused with physical inactivity (i.e. not meeting physical activity guidelines), but they are not synonymous. Rather it refers to the time spent sitting, lying or reclining during waking time and includes activities such as watching television, passive transportation (e.g. riding in a bus, train or car) and using a computer at a traditional desk [3]. Sedentary behaviour is largely recognized as an independent risk factor for chronic disease and mortality [4].

Canada has a long history of reporting on physical (in)activity [5]. Data on physical inactivity and sedentary behaviour are collected using various tools on several Statistics Canada health surveys including the Canadian Health Measures Survey (CHMS), the Canadian Community Health Survey (CCHS) and the Canadian Health Survey on Children and Youth. The Public Health Agency of Canada (PHAC) includes physical (in)activity as part of its national surveillance strategy around risk factors for non-communicable diseases using data from the CHMS and CCHS. These data have been, and continue to be, used to inform policy development and strategies around healthy living, with screen time being a specific sedentary behaviour of focus in the Canadian 24-Hour Movement Guidelines for the Early Years [6] and Children and Youth [7], ParticipACTION’s Report Card [8], and Canada’s Common Vision for Increasing Physical Activity and Reducing Sedentary Living [9]. These combined initiatives have helped to place Canada at the forefront of leaders in the field of sedentary behaviour. Canada continues to push boundaries in this area and is working towards improving its surveillance data on sedentary behaviour.

Alongside the surge in sedentary behaviour research, there was growing recognition within the PHAC and Statistics Canada, from both a policy and surveillance perspective, that there was a need to move beyond simply describing physical inactivity as it related to Canadians who did not meet physical activity guidelines. Rather, concepts such as sedentary time (including screen time), light intensity physical activity, and sleep, alongside their determinants and positive and negative health benefits, were important behaviours to include. It was essential to move beyond measuring adherence to physical activity guidelines [6, 7, 10] alone, and begin to examine and understand other intensities of movement during the rest of the day. From a surveillance perspective, it was important to understand how we might define these behaviours and their key components (e.g. screen time, reading time, non-active travel) accurately and measure them consistently over time at the population level. The growing public health need to monitor and report on these behaviours and related factors led to a modernization of the PHAC’s physical activity surveillance system which is now directed by the Physical Activity, Sedentary behaviour and Sleep (PASS) Indicators [11].

With the development of the PASS Indicators, it was clear that the PHAC had many ready-made indicators for physical activity, but that sedentary behaviour and sleep had fewer existing indicators to draw upon. Even with the long history of measuring screen time within Canadian national health surveys, it was clear from consultations between the PHAC and Statistics Canada that there was a need to update indicators and their associated measures to keep up with the sedentary practices of Canadians (especially around screen time consumption) and to consider sources of sedentary time beyond screen usage (e.g., commuting, sitting at work etc.) which were emerging in the scientific literature. For example, the original measures of screen time on earlier cycles of the CCHS did not include advances such as tablets and the use of social media on phones [12]. While efforts had been made over time to keep up with these societal changes in the CCHS and CHMS questions, ultimately it became clear that a more focused approach based on a thorough examination of the scientific literature and international survey best practices was warranted.

While survey methodology is often part of formal education, applying these methods in ‘real-world’ settings can be challenging. Further, the development process of national survey content, as well as how it is established is rarely described. We felt that presenting the methods undertaken in the development of this module would be of interest to those learning and applying these methods in other countries, contexts or towards other issues. In the case of surveys, content and questions must be based on the best available evidence, be valid and reliable, be relevant, have a high enough prevalence to be measurable with the survey’s sample size, and address important priorities. In the case of Canadian national surveys, they also need to be concise due to limited space and time and understandable for the general public to ensure quality responses. Understanding these factors is important for meeting the needs of national surveillance systems. The objective of this paper is to outline the process that the PHAC and Statistics Canada took in partnership with academic experts to develop a short, flexible, sedentary behaviour module for use on a national population survey (the CHMS) in order to improve the accuracy and reporting of sedentary behaviour in Canada. By outlining the development process of the sedentary behaviour module, the authors hope to provide important context and a framework for future module development and for those interested in using the module.

## Methods

**Fig. 1:**
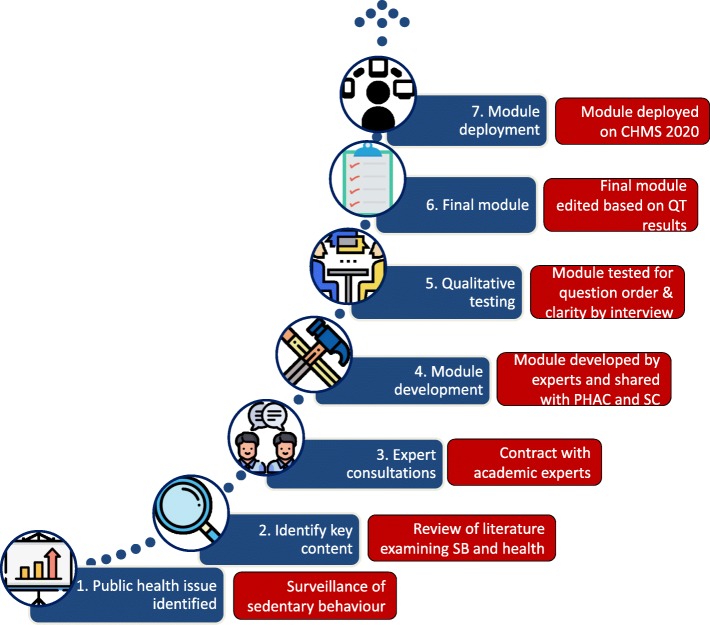
Process used for the development of a new sedentary behaviour module for a national health survey. CHMS – Canadian Health Measures Survey, PHAC – Public Health Agency of Canada, QT – qualitative testing, SB – sedentary behaviour, SC – Statistics Canada

Development of the sedentary behaviour module followed a multi-step process. The results of this paper describe this process (as seen in Fig. 1) and present a framework for survey content development.

## Results

### Understanding the evidence base

With the content of the 2020 cycle of the CHMS up for review, an opportunity to examine and revise sedentary behaviour content presented itself. To begin with, a contract was established between the PHAC and a leading expert in the field of sedentary behaviour (TJS) to provide a formal report on: 1) the evidence around specific types of sedentary behaviour (e.g. total sedentary time, screen time, reading, passive travel, etc.) related to health outcomes in order to provide context for question development and prioritization; and, 2) suggestions for a short sedentary behaviour module focusing on the most reliable and valid questions available from both the published literature, as well as national health surveys from around the world. Further instructions included that the module be developed to provide questions in order of prioritization with respect to their associated risks via the evidence-base. For example, if only one question could be posed, what would this look like or if only two questions could be posed, what would they include, etc.

The expert was free to involve or consult other experts as they thought appropriate and were encouraged to publish their findings. The contract resulted in a report back to the PHAC which included a proposed module, and subsequently, an article in a peer-reviewed, open-access journal describing the state of the evidence around the reliability and validity of self-report questions/questionnaires used in both research and national health surveys [13]. The expert report proposed a new sedentary behaviour module, entitled the International Sedentary Assessment Tool (ISAT) available for download here: http://www.sedentarybehaviour.org/sedentary-behaviour-questionnaires/. The module consisted of six sedentary questions supported by the literature; based on the most valid and reliable tools available. The questions were listed in order of prioritization with respect to their known association with health outcomes and provided an option for a single or two-item screen time question (depending on room on the survey). Finally the questions drew on current trends in sedentary behaviour including the importance of multi-tasking (e.g. sitting while watching TV and simultaneously using a smart phone) and changing types of screen media (e.g. tablets, smart phones, e-readers).

### Module development process

The module development process is outlined in Fig. 1. The expert report was shared with Statistics Canada in preparation for the 2020 CHMS consultations. PHAC and Statistics Canada held initial meetings to discuss the module questions proposed by the experts. Statistics Canada took the proposed module and modified language when appropriate (based on questionnaire development best practices [14]; see Table 1) and provided a draft of the module back to PHAC. PHAC was then able to propose edits and, where needed, consulted with experts in the field to ensure that the module items reflected the current state of scientific evidence and had appropriate face validity. Three versions of the questionnaire were drafted to ascertain whether the order of the questions impacted response and clarity, as well as to test the clarity of the single-item screen question compared to a two-part screen question.

**Table 1:** Sample of suggested survey design best practices and their application in the sedentary behaviour module development

Survey design best practice concept	Application to sedentary behaviour module
Identify all of the concepts to be measured	Identified and defined sedentary behaviour and its types and domains.
Modify writing of the questions to read at a grade 10 level	Adjusted previously validated questions to read at the grade 10 level by using appropriate language.
Cover only one topic/concept per question	Each question posed focuses on one type/domain of sedentary behaviour (e.g., sedentary time, screen time, travel, reading).
Break complex or conditional concepts into multiple questions	Recognizing that sedentary behaviour is engaged in differently during the week and weekend, each question includes a ‘per weekday’ and ‘per weekend day’ designation.
Phrase questions positively and avoid negative structures (i.e. don’t say: How often do you not go out because you cannot afford it? Better phrasing would be: How often do your finances affect your ability to go out?)	No negative structures are used in asking about time spent sedentary.
Avoid the use of leading or biased questions	There are no references to the sedentary behaviour of others in the questions.
Create a logical order and flow to ensure the process feels like an interview rather than an interrogation	We begin by asking respondents to estimate total sedentary time, and then ask about specific sedentary behaviours.
Design sensitive questions to ensure that their relevance is obvious or at least easy to explain	While none of the questions are ‘sensitive’, considerable thought was given to provide examples of pertinent activities under each question.
Keep the questionnaire as short as possible to ensure high response rates and to avoid partially completed interviews	The module was designed to be modular and as brief as possible. Each question can be asked on its own and questions can be added or dropped in response to the final estimate of available time for the survey.
Try to use consistent scales to avoid confusing respondents	Response options for all questions were standardized to reflect common formatting within the CHMS i.e. hours and minutes.

Once the module was thoroughly reviewed by the PHAC, it moved back to Statistics Canada for qualitative testing. The objectives of qualitative testing included: testing respondents’ understanding of specific concepts, terminology, questions and response categories; obtaining feedback from participants regarding their overall impression of and reactions to the questions; assessing respondents’ ability and willingness to respond to survey questions; determining the appropriateness and completeness of the response categories; testing the respondent-friendliness of the questions (i.e. they are easily understood and accurately completed); and, estimating how much time the module takes to administer.

Qualitative testing was undertaken by the Questionnaire Design Resource Centre (QDRC) at Statistics Canada and included 21 one-on-one in-depth interviews; 11 English and 10 French. One observer from the questionnaire development team was physically present at each interview while other project team members observed the interviews remotely using Cisco WebEx (online meeting/video conferencing software) and provided real-time questions to the moderator. Interviews lasted no more than one hour and respondents received a monetary compensation at the end. Participants were recruited based on a variety of factors and identified: youth aged 12–17; parents of children aged 3–11 years (proxy for child responses as per CHMS methodology); people who had broken or fractured bones; people with arthritis or osteoporosis; and, those who spent time in the sun in the past two months. The last three categories were chosen to accommodate other modules that were simultaneously tested.

Results of the qualitative testing were summarized in a report from the QDRC and distributed to the project team. The report identified areas of concern via the respondents for the three tested questionnaire modules. With respect to the total sitting time question, qualitative testing revealed that respondents often excluded sitting time that occurred in domains outside of leisure time (e.g. travel, work/school, household) and therefore, total sitting time was likely under-reported. However, it was noted that placing the total sitting time question following the sub-domain questions (screen, reading, transport) led to respondents recalling only sitting time that occurred outside of the other domains. Respondents largely commented that the week and weekend day delineation was important given the changing nature of their behaviours based on the type of day; however, participants who were retired commented that sitting time was similar regardless of week/weekend status. Many respondents identified that it was not always clear that they were being asked to report daily totals versus weekly totals. Most respondents found it easier to be asked a single screen time question versus two which separated out TV from computer-type screen time. Parents who proxy-reported sitting time for their children reported it was difficult to report time spent sitting during school hours.

**Fig. 2:**
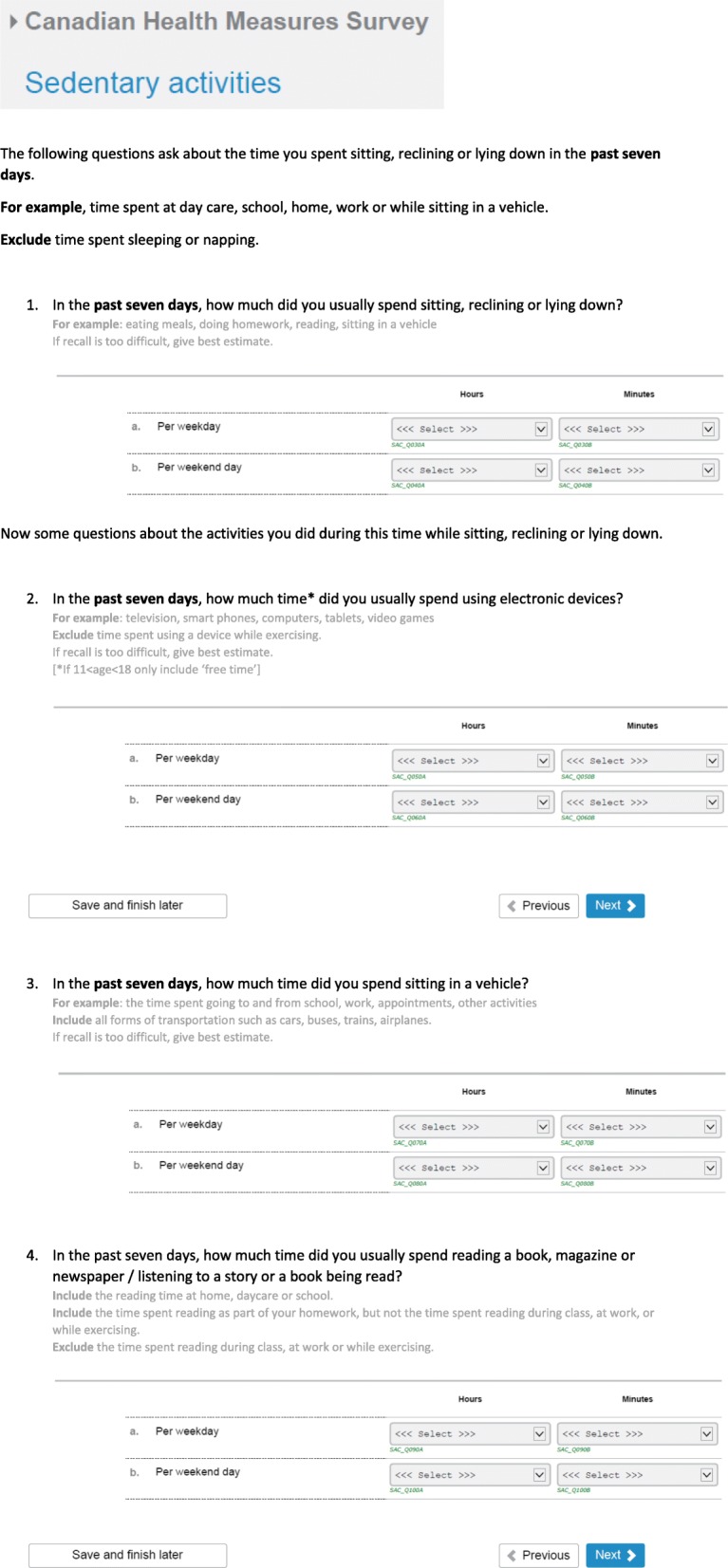
Draft sedentary behaviour module

Results of the qualitative testing were used to revise the sedentary module. Based on feedback from respondents, the following changes were made: 1) the lead-in for all of the questions was changed to include “In the past **7 days,** how much time **per week day** …” ; 2) the total sedentary time question was rephrased to also include sitting in a vehicle as an example; and, 3) a single screen-time question was chosen as it was most clear for respondents. A draft of the module can be found in Fig. 2.

## Discussion

This paper describes the rigorous process that the PHAC and Statistics Canada undertook to develop a new sedentary behaviour module for the CHMS (and future population-based surveys). The number of questions in the module is limited due to time constraints given that the CHMS collects information on many factors related to the health and health habits of Canadians (i.e. there is a limited amount of time allotted for asking questions to reduce respondent burden and maintain response rates). Due to these time constraints, other types and domains of sedentary behaviour such as workplace sedentary time were not included in the final module; the evolution of evidence will likely bring suggestions for future revisions and it is important that these changes (e.g., workplace sedentary time) be considered using a similar development process.

We recognize that validation of the module is important once data is collected. It is essential that as new modules are developed and used in surveys that their reliability and validity are examined to evaluate their performance and that they are refined accordingly. The CHMS collects objectively measured movement data using accelerometers and thus offers the opportunity for future comparisons between self-reported sedentary behaviours (i.e., screen time, sitting time) and accelerometer-measured sedentary time. It is important to stress that while comparisons are possible, validation of this module’s sedentary behaviour questions is not. Accelerometers capture total sedentary time but lack any information about the context of the sedentary behaviours. In contrast, this questionnaire estimates time spent in specific sedentary pursuits (e.g., screen time) but is limited in its ability to capture every minute of sedentary behaviour incurred throughout the day. This incongruence between two measures of the same construct is a limitation and challenge of measuring health behaviours. We will assess the criterion or predictive validity, examining how these measures associate with commonly known health outcomes (e.g., cardiovascular disease, diabetes). With continued use, we will also be able to assess the reliability of the measure.

## Conclusions

Currently many developed countries are seeking solutions to the phenomena of declining response rates on national surveys [15]. One solution has been to shorten surveys to render them less time consuming; inevitably resulting in a greater demand for survey space. The process that created the ISAT presents a practical solution to this epidemiological phenomenon, which like many challenges, also brings an opportunity. In this case it required a greater emphasis on: thinking about the module in the preplanning phase of the survey; building in flexibility and ranked priorities into the module; and, basing the development and process on the best science and international best practices. For Statistics Canada this collaborative effort reflected their increased attention towards three key pillars of their modernization strategy including: user-centric delivery; leading-edge methods and sharing; and, collaboration. For the PHAC, the collaboration produced a documented process on the development of the sedentary behaviour module used in the CHMS and a framework for future module development (Fig. 1). This paper’s description of how the ISAT was subsequently modified for inclusion on a national health survey will hopefully provide other potential users with the information required to incorporate this module into their own survey instruments, as well as a process they might use for their own future module development.

## Data Availability

Not applicable.

## Abbreviations

CCHS: Canadian Community Health Survey
CHMS: Canadian Health Measures Survey
ISAT: International Sedentary Assessment Tool
PASS: Physical Activity, Sedentary behaviour and Sleep
PHAC: Public Health Agency of Canada
QDRC: Questionnaire Design Resource Centre
